# Novel attentional gait index reveals a cognitive ability-related decline in gait automaticity during dual-task walking

**DOI:** 10.3389/fnagi.2023.1283376

**Published:** 2024-01-11

**Authors:** Shuqi Liu, Andrea L. Rosso, Emma M. Baillargeon, Andrea M. Weinstein, Caterina Rosano, Gelsy Torres-Oviedo

**Affiliations:** ^1^Sensorimotor Learning Laboratory, Department of Bioengineering, University of Pittsburgh, Pittsburgh, PA, United States; ^2^Center for the Neural Basis of Cognition, Pittsburgh, PA, United States; ^3^Department of Epidemiology, University of Pittsburgh, Pittsburgh, PA, United States; ^4^Department of Medicine, Division of Geriatric Medicine, University of Pittsburgh, Pittsburgh, PA, United States; ^5^Department of Psychiatry, School of Medicine, University of Pittsburgh, Pittsburgh, PA, United States

**Keywords:** aging, near-infrared spectroscopy, locomotion, community mobility, cognition, brain imaging, motor control, interference

## Abstract

**Introduction:**

Gait automaticity refers to the ability to walk with minimal recruitment of attentional networks typically mediated through the prefrontal cortex (PFC). Reduced gait automaticity (i.e., greater use of attentional resources during walking) is common with aging, contributing to an increased risk of falls and reduced quality of life. A common assessment of gait automaticity involves examining PFC activation using near-infrared spectroscopy (fNIRS) during dual-task (DT) paradigms, such as walking while performing a cognitive task. However, neither PFC activity nor task performance in isolation measures automaticity accurately. For example, greater PFC activation could be interpreted as worse gait automaticity when accompanied by poorer DT performance, but when accompanied by better DT performance, it could be seen as successful compensation. Thus, there is a need to incorporate behavioral performance and PFC measurements for a more comprehensive evaluation of gait automaticity. To address this need, we propose a novel attentional gait index as an analytical approach that combines changes in PFC activity with changes in DT performance to quantify automaticity, where a reduction in automaticity will be reflected as an increased need for attentional gait control (i.e., larger index).

**Methods:**

The index was validated in 173 participants (≥65 y/o) who completed DTs with two levels of difficulty while PFC activation was recorded with fNIRS. The two DTs consisted of reciting every other letter of the alphabet while walking over either an even or uneven surface.

**Results:**

As DT difficulty increases, more participants showed the anticipated increase in the attentional control of gait (i.e., less automaticity) as measured by the novel index compared to PFC activation. Furthermore, when comparing across individuals, lower cognitive function was related to higher attentional gait index, but not PFC activation or DT performance.

**Conclusion:**

The proposed index better quantified the differences in attentional control of gait between tasks and individuals by providing a unified measure that includes both brain activation and performance. This new approach opens exciting possibilities to assess participant-specific deficits and compare rehabilitation outcomes from gait automaticity interventions.

## 1 Introduction

The ability to move around in the community is essential for independent living (Patla and Shumway-Cook, 1999; Rosso et al., 2013; Sheppard et al., 2013; Rantakokko et al., 2016). Successful community mobility requires gait automaticity (Van Swearingen and Studenski, 2014; Clark et al., 2014b; Brustio et al., 2018), which refers to the automatic control of walking with minimal recruitment of attentional networks primarily residing in the prefrontal cortex (PFC) (Van Swearingen and Studenski, 2014; Clark, 2015). Gait automaticity declines with age (Gschwind et al., 2010; Montero-Odasso et al., 2011; Van Swearingen and Studenski, 2014; Clark, 2015) and may contribute to an increased risk of falls (Gschwind et al., 2010; Montero-Odasso et al., 2011). Therefore, it is important to have a standardized way to quantify gait automaticity to help evaluate fall risks and promote mobility in older adults.

Gait automaticity can be evaluated by behavioral or neurophysiological assessments. The behavioral assessment uses dual-task walking (DT, i.e., walking and performing a cognitive task simultaneously) and compares the performance in DT relative to single-task (ST, i.e., either walking or performing a cognitive task) (Paul et al., 2005). High automaticity is reflected by similar walking (e.g., gait speed or task completion time) and cognitive performance in DT and ST (Paul et al., 2005; Brauner et al., 2021; Longhurst et al., 2022). When walking is automatic, it is assumed to require minimal attentional resources, resulting in no impact on the participant’s performance on the cognitive task (Clark, 2015). The neurophysiological approach more directly measures the attentional resources required to complete the task. The attentional resources are usually quantified by the cortical activation of the PFC (Holtzer et al., 2011; Clark, 2015; Herold et al., 2017; Menant et al., 2020) using non-invasive brain imaging technologies such as functional near-infrared spectroscopy (fNIRS).

DT paradigms integrated with fNIRS-based PFC measurements provide a useful avenue to assess gait automaticity. However, it is challenging to interpret PFC measurements and task performance independently during automaticity assessments. Specifically, a small change in PFC activation from ST to DT has opposing interpretations depending on task performance. Namely, a small increase in PFC activation alongside good task performance may indicate high automaticity (Beurskens et al., 2014; Clark, 2015; Mirelman et al., 2017). In contrast, when a small increase in PFC activation is coupled with poor task performance, it could indicate inefficiency in recruiting neural resources or the task is beyond capacity (Holtzer et al., 2011; Beurskens et al., 2014), as suggested by the CRUNCH model (Reuter-Lorenz and Cappell, 2008). Similarly, a large increase in PFC activation from ST to DT yields different interpretations contingent on task performance. A larger increase in PFC activation paired with maintained task performance could imply successful compensation (Clark et al., 2014b; Holtzer et al., 2015; Mirelman et al., 2017) from PFC for other brain regions whose structures and integrities have declined with aging, as suggested by several theories such as the HAROLD and STAC models (Cabeza, 2002; Reuter-Lorenz and Cappell, 2008; Park and Reuter-Lorenz, 2009; Maillet and Rajah, 2013; Fettrow et al., 2021). Conversely, when a large increase in PFC activation is paired with poor task performance, it could represent unsuccessful compensation (Clark et al., 2014a; Fraser et al., 2016; Osofundiya et al., 2016; Mirelman et al., 2017) or dedifferentiation, i.e., non-task-specific overactivation (Cabeza, 2002; Cabeza and Dennis, 2012; Festini et al., 2018; Fettrow et al., 2021). Therefore, there is a need to consider PFC activation and task performance simultaneously to characterize gait automaticity more accurately.

Given the interrelation of PFC activation and behavioral performance, analyzing them together may provide a more comprehensive measure of an individual’s gait automaticity than examining either alone. To address this need, we propose a novel attentional gait index that combines PFC activity and DT performance to quantify gait automaticity. Specifically, a large index represents a greater need for attentional control during walking when automaticity is comprised. The objective of the study was to test the ability of the index to differentiate the change in automaticity (1) between tasks within the same participants and (2) between participants. For objective 1, to compare between tasks, we computed the attentional gait index in older participants ( ≥ 65 years of age, *n* = 173) who completed two difficulty levels of DT. We expected a higher attentional gait index, indicating worse automaticity, at greater levels of task difficulty. For objective 2, to compare between participants, we tested if an individual’s cognitive abilities, measured by Mini-Mental State Exam scores, were related to automaticity. We anticipated that better cognitive ability would be correlated with a lower attentional gait index (i.e., better automaticity) and that associations would be stronger for the attentional gait index compared to either behavioral or neurophysiological measures alone.

## 2 Materials and methods

### 2.1 Participants

The data included participants from three previously published studies: Program to Improve Mobility in Aging, *n* = 42 (Brach et al., 2020); Neural Mechanisms of Community Mobility, *n* = 29 (Aizenstein et al., 2008); and Move Monongahela-Youghiogheny Healthy Aging Team, *n* = 102 (Ganguli et al., 2010). The three datasets, although collected for different purposes with different experimenters, included similar protocols. Two studies were collected in the same lab with the same fNIRS system, while the other study was collected in a different laboratory with different fNIRS equipment (same model). All data collection was overseen by the same investigator. There was no statistical difference by study of PFC activation or change in performance from ST to DT. Therefore, all datasets were combined for analysis.

All study participants were at least 65 years old, able to walk unassisted, fluent English speakers, had no dementia, had no severe vision or hearing impairment, and had no major neurological or psychiatric diseases. More study-specific inclusion criteria and medical conditions were reported in the published studies (Aizenstein et al., 2008; Ganguli et al., 2010; Brach et al., 2020). The Institutional Review Board at the University of Pittsburgh approved the studies, and all participants gave written informed consent.

#### 2.1.1 Participant characteristics

In all studies, age, sex, race, and highest level of education were self-reported. General cognitive ability was assessed using the Mini-Mental State Exam (MMSE), a commonly used screening instrument for cognitive impairments (Folstein et al., 1975). The Program to Improve Mobility in Aging study administered the Modified Mini-Mental State (3MS) Examination (Teng and Chui, 1987), which is an expanded version of the MMSE and includes all items of MMSE (Van Patten et al., 2019). For consistency across studies, MMSE scores were derived from the 3MS in this study by extracting only items equivalent to the MMSE.

### 2.2 Experimental paradigm

**Figure 1:**
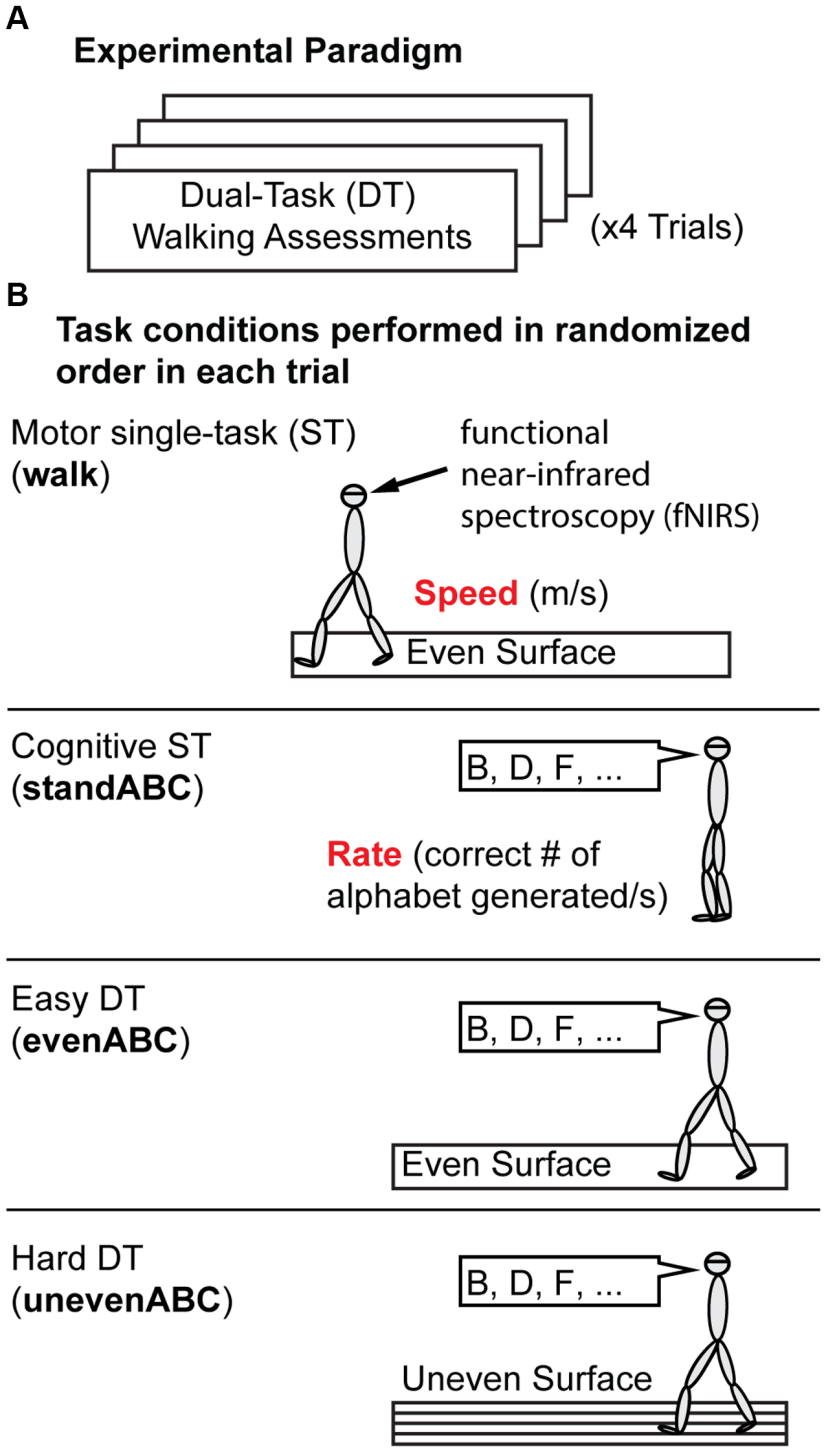
Experiment Protocol. **(A)** Overall protocol. Participants performed four trials of dual-task (DT) assessments. **(B)** Each trial includes four task conditions presented in pseudo-random order. The four tasks are a motor single-task (ST): walking on an even surface; a cognitive ST: standing and reciting every other letter of the alphabet (standABC); and two DTs: reciting every other letter of the alphabet while walking on an even (evenABC) or uneven (unevenABC) surface. Motor performance is measured by gait speed (m/s), and cognitive performance is measured by the rate of correct letters generated. Prefrontal cortex activation was measured by functional near-infrared spectroscopy throughout the trial.

Participants performed single or dual tasks on an oval track with a 15-meter straight walkway on each side. One side had a standard surface (even), and the other side had wood prisms underneath carpets (uneven; Thies et al., 2005; Hoppes et al., 2020). All participants performed four experimental trials, and each trial included a pseudo-randomized sequence of four conditions outlined here: two single tasks (ST) and two dual tasks (DT) (Figure 1). The order was pseudo-randomized such that walking continued along the oval track. Single tasks included a motor single-task, walking at a comfortable pace on an even surface (walk), and a cognitive single-task, standing while reciting every other letter of the alphabet starting from B (standABC; Holtzer et al., 2011; Brandler et al., 2012; Verghese et al., 2012). The single tasks were used as a reference to compute the change in performance during DT to account for the different baseline abilities of each participant. The DT conditions required performing the cognitive task while walking on even (evenABC) or uneven surfaces (unevenABC). No instruction was given about which task to prioritize. The unevenABC condition was considered a harder DT condition than the evenABC because of the increased challenge of balancing and walking on the uneven floor surface.

The task duration was 20 s for standABC. The duration for the motor ST (walk) and both DTs (evenABC and unevenABC) varied depending on the time the participant took to walk over the 15 m straightway. Every condition was preceded by a quiet standing for 20 s where participants were simply instructed to stand still and not do anything. The quiet standing allowed the hemodynamic response to return to rest level such that relative changes in oxygenation of the blood could be computed for each task condition compared to quiet standing.

### 2.3 Data collection

Motor performance was quantified by gait speed (m/s). Gait speed is computed as the distance (15 m) divided by the time it took to walk the 15-meter walkway, where time was measured by a stopwatch. Cognitive performance was quantified by the rate of correct letters of the alphabet generated per trial duration (correct letters/s). Average motor and cognitive performance for each condition across the four trials is reported.

PFC activation was measured using fNIRS, which measures changes in blood oxygenation based on the distinct light absorption properties of oxygenated (Hbo) and deoxygenated (Hbr) hemoglobin (Miyai et al., 2001; Perrey, 2014; Hoppes et al., 2020). Participants wore an eight-channel continuous wave fNIRS headband (Octamon, Artinis Medical Systems, Netherlands) over their forehead during the entire experiment. The headband contained two detectors and eight sources with a source and detector pair distance of 35 mm covering both the left and right PFC regions, specifically Brodmann areas 9, 44, 45, and 46 (Hoppes et al., 2020). The center of the headband was aligned with the center of the participant’s nose, and the bottom of the headband was just above the eyebrow (Bohlke et al., 2023). No short separation channel was available in the equipment, and physiological and extracortical noises were addressed statistically (See 2.4 fNIRS Data Analysis). Near-infrared light transmitted at 850 nm and 760 nm was used to detect changes in Hbo and Hbr. Data were sampled at 10 Hz and collected by the OxySoft software (Artinis Medical Systems, Netherlands).

### 2.4 fNIRS data analysis

fNIRS data were processed using the NIRS Brain AnalyzIR toolbox (Santosa et al., 2018) in MATLAB 2021b (Mathworks, Natick, Massachusetts). Data for a given task were excluded from processing and analysis (1.51% of the tasks) if there were experimental errors or if the participant clearly violated the protocol (e.g., walking during a standing task). During processing, the fNIRS recording for each trial was first trimmed to keep only 2 s of the data before and after the first and last task of a trial to reduce global baseline noise. Data channels with flat signals due to data saturation or equipment malfunction were identified by flagging channels with small variance (less than 10^−9^ in a 5-s moving variance window). The flat channels (0.97% of data) were then visually inspected and removed from analysis. Light intensity was converted to optical density and then converted to Hbo and Hbr measurements using the modified Beer–Lambert law with a partial path length factor of 0.1 (Hoppes et al., 2020). The time series data for each source–detector pair was used to fit a general linear model (GLM). The design matrix of the GLM was the convolution of stimulus timing, duration, and a canonical hemodynamic response function (Hoppes et al., 2020). To minimize motion and physiological artifacts, the model was solved with an autoregressive pre-whitening iteratively reweighted least square approach (Santosa et al., 2018). In brief, the autoregressive filter is a statistical method to alleviate physiological noise and motion artifacts. The iterative reweighted least square approach further downweights large motion artifacts (Barker et al., 2013; Santosa et al., 2018; Bohlke et al., 2023). A Student’s *t*-test was then performed on the regression coefficients, and the *t*-score represents the changes in Hbo or Hbr in each task compared to the quiet standing before the task. The results across four trials for the eight channels covering the whole PFC were combined, taking into account the covariance using the toolbox to generate one ΔHbo and one ΔHbr for each participant for each task relative to quiet standing. Typically, an increase in PFC activity will be represented by an increase in oxygenated hemoglobin (i.e., a positive ΔHbo value) and a decrease in deoxygenated hemoglobin (i.e., a negative ΔHbr value).

ΔHbo often has a stronger signal-to-noise ratio (Menant et al., 2020) and, therefore, was used as the main measure of PFC activation for the remainder of the article. However, the same approach applies to both Δbo and ΔHbr measurements. The results of calculating the index using Hbr are included in Supplementary materials.

### 2.5 Attentional gait index

The goal of the attentional gait index is to create a single monotonic axis that combines performance and PFC activation, where a larger index will always represent a greater need for attentional control during walking, indicating worse automaticity. Worse automaticity is reflected by more interference between dual tasks where they compete for the same attentional resources (Paul et al., 2005), leading to decreased task performance and increased recruitment of attentional resources (i.e., higher PFC activation).

The attentional gait index combining cortical activation and performance is defined as the following:


(1) $$\mathrm{Attentional}\;\mathrm{Gait}\;\mathrm{Index}=\mathrm{Gain}*PFC_{\mathrm{Activation}}$$

where $\mathrm{Gain}=f\left(\Delta \mathrm{Performance}\right)=e^{-\alpha *\Delta \mathrm{Performance}}$

α= hyperparameter regulating the impact of ΔPerformance on the Attentional Gait Index


$$\Delta \mathrm{Performance}=\frac{\Delta \mathrm{Motor}}{\mathrm{Moto}r_{ST}}+\frac{\Delta C\mathrm{ognitive}}{\mathrm{Cognitiv}e_{ST}}=\frac{\mathrm{Spee}d_{DT}-\mathrm{Spee}d_{ST}}{\mathrm{Spee}d_{ST}}+\frac{\mathrm{Rat}e_{DT}-\mathrm{Rat}e_{ST}}{\mathrm{Rat}e_{ST}}$$

ST = single-task conditions (walk or standABC)

DT = dual-task conditions (evenABC or unevenABC)

Speed = gait speed (m/s), measures motor performance

Rate = rate of correct letters of alphabet generated (letters/s), measures cognitive performance.

**Figure 2:**
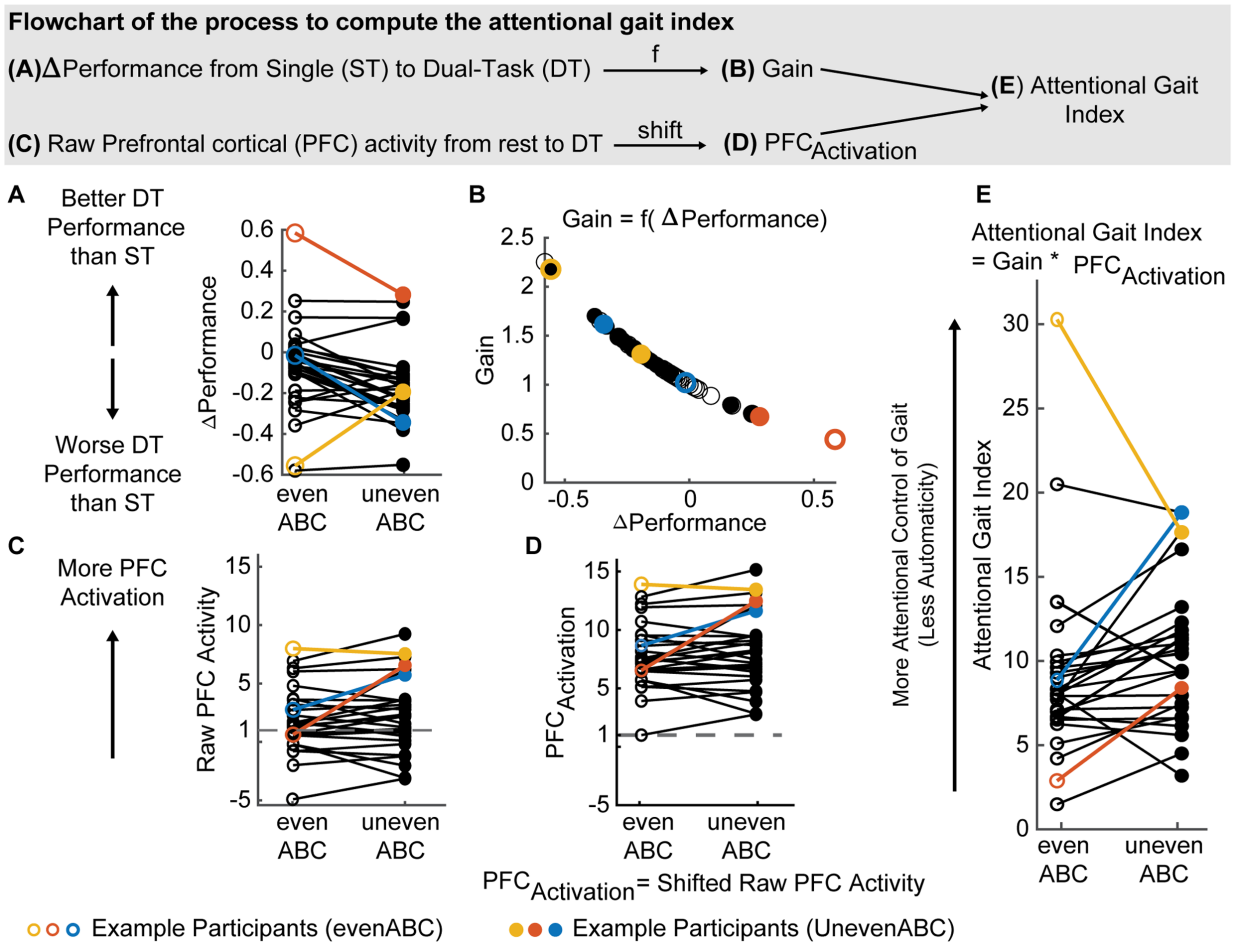
Schematics and flowchart illustrating the process of computing the attentional gait index. The displayed example is one of the included datasets (*n* = 29) with three example participants highlighted (yellow, red, and blue). The evenABC (easier dual task, DT) task is represented by hollow circles, and the unevenABC task is marked with filled circles. **(A)** Normalized performance change from single-task (ST) baseline to each DT. The performance combines both motor and cognitive performances. A positive value indicates performance is better in DT than in ST. **(B)** Calculation of the gain from ΔPerformance. A large decrease in performance is mapped to a large gain (e.g., yellow empty circle). **(C)** The raw measure of the prefrontal cortex (PFC) activity, which are t-scores representing changes in oxygenated hemoglobin (ΔHbo) concentration from rest to each DT. A positive value indicates increased Hbo concentration in DT compared to quiet standing. A negative value indicates a decrease in Hbo concentrations in DT from rest. **(D)** The second term in the attentional gait index equation, PFC_Activation_, simply shifts raw PFC activity values to be above or equal to 1. **(E)** The computed attentional gait index, which is the multiplication of the gain **(B)** and the PFC Activation **(D)**.

The attentional gait index is generated by scaling PFC_Activation_ with a gain that is a function of the hyperparameter, α , and performance change, ΔPerformance (Eq. 1). Figure 2 demonstrates the process of computing the attentional gait index using one of the datasets with three example participants highlighted to visualize the transformation of the data.

The performance change, denoted as ΔPerformance (Figure 2A), is computed as the combined changes in cognitive and motor performances from ST to DT, normalized to performance in domain-specific ST (standABC and walk, respectively, Eq. 1). The normalization accounts for individual differences in walking speed and alphabet performance at baseline. Cognitive and motor performances are weighted equally in ΔPerformance to account for the different strategies employed by the participants during dual tasking since no specific prioritization instruction was given (Verghese et al., 2007; Yogev-Seligmann et al., 2010; Laguë-Beauvais et al., 2015). Studies with prioritization in the instruction should consider weighting the motor and cognitive performance differently. The definition of ΔPerformance is similar to dual-task cost in existing literature (Brauner et al., 2021; Longhurst et al., 2022). A more negative ΔPerformance value indicates performance was worse (i.e., participants walked slower and/or generated fewer correct alphabet letters) during DT compared to ST. Conversely, a positive ΔPerformance represents better performance in DT compared to ST.

A negative sign was added before ΔPerformance (Eq. 1) because we expected performance to decrease during DT (i.e., ΔPerformance ≤ 0; Figure 2A, majority of data points < 0). The negative ΔPerformance reflects cognitive–motor interference during DT, which is expected in populations where gait automaticity is reduced, such as older adults.

The exponential weighting in the gain (Eq. 1) is chosen such that (1) performance decreases (ΔPerformance < 0) scale the attentional gait index up, with a large decrease weighted much more than a small decrease (Figure 2B), (2) performance improvements (ΔPerformance > 0, less common occurrences; Figure 2B) scale the attentional gait index down, but to a much smaller extent, and (3) unchanged performance will result in an index that is equal to the PFC activation. We weighted the gain in this way so that the index would more sensitively discriminate between participants with decreased rather than increased DT performance, as decreased performance with increasing task challenge is expected and is particularly relevant in the participant populations where gait automaticity is most often studied.

The hyperparameter α in the gain equation regulates the impact of ΔPerformance on the attentional gait index (Eq. 1). α was optimized to minimize outliers while maximizing the sensitivity to the increase in attentional control of gait (i.e., reduction in gait automaticity) as the task difficulty increased from the evenABC to the unevenABC task. Specifically, the objective function is defined as:


(2) $$\begin{array}{l} \mathrm{Objective}\;\mathrm{function}=\%\left(\mathrm{Attentional}\;\mathrm{Gait}\;{\mathrm{Index}}_{\mathrm{hardDT}}>\mathrm{Attentional}\;\mathrm{Gait}\;{\mathrm{Index}}_{\mathrm{easyDT}}\right)-\%\mathrm{outlier}\\ \;=\%\left(\mathrm{Attentional}\;\mathrm{Gait}\;{\mathrm{Index}}_{\mathrm{unevenABC}}>\mathrm{Attentional}\;\mathrm{Gait}\;{\mathrm{Index}}_{\mathrm{evenABC}}\right)-\%\mathrm{outlier} \end{array}$$

Outliers were defined as values more than three scaled median absolute deviations from the median, which is a robust measure of dispersion and outliers (Leys et al., 2013). The objective function value was evaluated for $\alpha \in \left[0,5\right]$ with 0.1 increments. The optimal α was chosen as the smallest α that maximizes the objective function. At the start of the search range ( α=0 ), the gain from ΔPerformance is equal to 1 and the attentional gait index is equal to the PFC activation, which is the current standard in the field. The upper bound of the search range α= 5 was chosen heuristically for the combined dataset. Notice that the search range and optimal α value found are specific to the current dataset, but the objective function (Eq. 2) and the optimization procedure can be applied to any experimental design with two levels of DT difficulties.

To maintain the monotonic property of the attentional gait index and the relative differences across people and conditions, we turned all PFC_Activation_ values positive by shifting all PFC activity data, i.e., ΔHbo at DT compared to ST, by an offset (Figures 2C,D), specifically:


(3) $$\begin{array}{l} PFC_{\mathrm{activation}}=f\left(\mathrm{Raw}\;PFC\;\mathrm{Activities}\right)\\ \;=f\left(\Delta Hbo\right)=\Delta Hbo+\min\left(\Delta Hbo\right)+\varepsilon ,\\ \;\mathrm{where}\;\varepsilon >0 \end{array}$$

We shifted all PFC activation values by the minimum value across participants and tasks and then included an offset ( ε=1) such that the minimum PFC activation would be different than zero (Eq. 3). This was done to keep the attentional gait index (the scaled PFC activation) monotonic while maintaining the relative difference in PFC activation across participants. Notice that Eq. 3 is a linear operation. Thus, the specific value of ε does not impact the results. In this case, we chose ε = 1 for simplicity (Figure 2D). Since the shift eliminates the sign of Hbo changes, our measure cannot determine how the attentional gait index changes with respect to rest. However, it maintains the relative differences between tasks within a participant and across participants. In other words, the linear operation does not impact the goal of the attentional gait index, which is to compare attentional control of gait (i.e., a proxy for gait automaticity) across tasks and individuals. A higher value before or after the shifting will always represent more PFC activation during the task.

If we were to use Hbr to represent the PFC activation, Hbr is the first sign flipped to maintain the convention where higher $PFC_{\mathrm{Activation}}$ always represents more PFC activity (see Supplementary materials).

Figure 2 demonstrates the flowchart for computing the attentional gait index using one of the three included datasets. The blue participant is a typical example that behaves as we expected from the task design. As task difficulty increased, the blue participant’s performance decreased (Figure 2A, negative slope) and PFC activation increased (Figures 2C,D, positive slope), suggesting the harder task had more interference between walking and the alphabet tasks and required more attentional resources. In other words, the harder task was performed with higher attentional control, i.e., less automaticity. As a result, the harder task had a larger attentional gait index (Figure 2E, positive slope). In contrast, the yellow participant increased performance (Figure 2A) and decreased PFC activation (Figures 2C,D) as the task became harder, suggesting the harder task was performed with improved automaticity (Figure 2E, lower value in attentional gait index for the harder task). This could result from the participant not being fully engaged in the easy task or the overall task design not being challenging enough.

The attentional gait index is also effective in differentiating between participants. The red and blue participants had similar patterns of performance change (Figure 2A, negative slopes for both) and PFC activation (Figures 2C,D, positive slopes), but the red participant achieved better performance than the blue participant (ΔPerformance red > blue in both tasks). This observation suggests that with similar PFC resources, the red participant utilized the resources more effectively to achieve better performance. Consequently, the red participant should be considered to have better automaticity than the blue participant, i.e., lower attentional gait index values (Figure 2E, red much smaller than blue).

### 2.6 Statistical analysis

For objective 1, to assess how each metric captured the anticipated task-difficulty changes in gait automaticity, we descriptively compared the attentional gait index, PFC activation, and ΔPerformance by reporting the percentage of participants following the expected trend for each metric as task difficulty increases.

For objective 2, linear regressions were performed to test if the independent variable, MMSE scores, is related to the dependent variables, attentional gait index, PFC activation, and performance, while adjusting for demographics: age, sex, race, and highest level of education. Since the variables are on different scales, all variables were first standardized as Z-scores before the model fitting. The variance inflation factor (VIF) was computed to assess the multicollinearity among the independent variables in the adjusted models. Variables with VIF < 10 would be kept in the multiple regression models. Model significance was determined by an F-test comparing the regression model with a constant model. To compare the impact of MMSE across the models, the standardized β is reported. In brief, the standardized β represents in standard deviation unit how much a unit increase in MMSE will impact the dependent variable. We reported the ordinary *R*^2^ values, the *p*-values for the models, and standardized coefficient estimates with their respective standard errors and *p*-values. A one-sample Kolmogorov–Smirnov test was performed to check the normality of the standardized residuals.

Two participants did not follow the instructions to recite every other letter of the alphabet. A sensitivity analysis was included to test the impact of removing these two participants on the fitting of the models adjusted for demographics and our conclusions. All analyses were performed in MATLAB 2020a, and a statistical significance of α=0.05 was used.

## 3 Results

Demographics, cognitive test results, cognitive and motor task performances, and fNIRS measurements of PFC activation in dual tasks are shown for the full sample in Table 1. Among the 22 non-white participants, one participant identified as Asian, one identified as American Indian or Alaskan Native, 19 identified as Black, and one identified as mixed or other race. Among the participants with education less than or equal to high school, only one participant’s highest level of education was less than high school.

**Table 1:** Demographic, clinical assessment results, task performances, and prefrontal cortical (PFC) activation (ΔHbo) of the study participants.

Variables	Mean (SD) or n (%)
Sample size	173
Age (years)	73.9 ± 5.6
Female, n (%)	108 (62.4%)
Race, n (%)	
White	151 (87.3%)
Non-white	22 (12.7%)
Highest level of education, n (%)	
Less than or equal to high school/equivalent	43 (24.9%)
College	87 (50.3%)
Postgraduate	43 (24.9%)
Mini-Mental State Exam (MMSE) score (max 30)	28.4 ± 1.8
Gait speed at walk (motor single-task, m/s)	1.01 ± 0.17
Gait speed at evenABC (m/s)	0.90 ± 0.19
Gait speed at unevenABC (m/s)	0.84 ± 0.19
Rate of correct alphabet letters at standABC (cognitive single-task, letters/s)	0.56 ± 0.15
Rate of correct alphabet letters at evenABC (letters/s)	0.59 ± 0.16
Rate of correct alphabet letters at unevenABC (letters/s)	0.58 ± 0.16
Change in oxygenated hemoglobin (ΔHbo) from rest to evenABC (*t*-stats)	1.64 ± 2.85
ΔHbo from rest to unevenABC (t-stats)	1.99 ± 3.24

All data are shown as mean ± SD except for sex, which is binary, and race and highest level of education, which are categorical.

### 3.1 Attentional gait index increases as task difficulty increases

**Figure 3:**
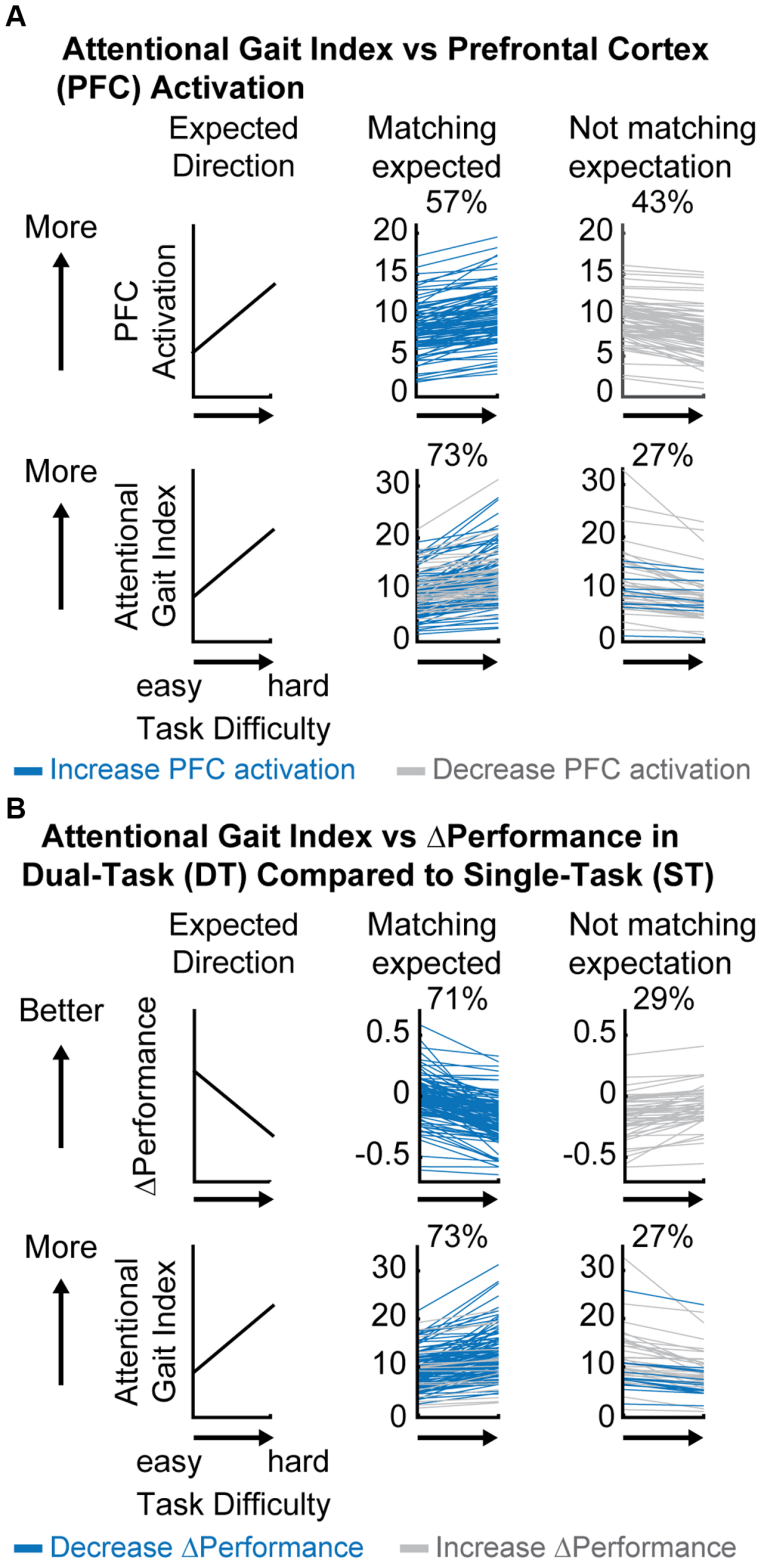
Descriptive comparison between attentional gait index, prefrontal cortical (PFC) activation, and ΔPerformance as task difficulty increases. **(A)** Comparison between attentional gait index and PFC activation at each dual-task (DT) relative to rest. Blue always represents participants who showed an expected increase in PFC activation (top middle panel) and gray always represents participants who decreased PFC activation (top right) as the task became more difficult. Colors in the bottom panel represent how participants from different groups at the top panel moved into different categories (increase or decrease) in attentional gait index. Notice that some participants with an unexpected decrease in PFC activation will now have an expected increase in attentional gait index after considering performance (gray in the top right moved to the bottom middle panel). **(B)** Comparison between attentional gait index and ΔPerformance. Blue always represents participants who showed an expected decrease in ΔPerformance as the task becomes harder (top middle panel) and gray always represents the ones who increased ΔPerformance (top right). Colors in the bottom panel represent how participants from different ΔPerformance at the top panel moved into different categories (increase or decrease) in attentional gait index (bottom panel).

By experimental design, a harder task should require more attentional resources, i.e., increased PFC activation (Figure 3A top left panel) and have poorer performance (Figure 3B top left panel), resulting in worse automaticity, i.e., increased attentional gait index (Figures 3A,B bottom left panels).

Only 57% of participants increased PFC activation as the task became harder (Figure 3A top middle panel), showing no clear evidence of increased attentional control as task difficulty increased by this measure alone. However, 73% of participants increased attentional gait index (i.e., had worse automaticity) as task difficulty increased (Figure 3A bottom middle panel), matching our anticipated result that more challenging tasks would be performed with lower automaticity. Notice that the increase from 57 to 73% of participants following our expectation was primarily due to the gray participants whose PFC activation unexpectedly decreased with increased task difficulty (Figure 3A top right). These gray participants recruited less PFC as the task became harder, but their performance declined, suggesting that PFC resources were not being used as effectively as needed by the task demand, which indicates reduced automaticity in the harder task.

In comparison, a larger percentage of participants (71%) decreased their performance (combined cognitive and motor performance) as the task became harder (Figure 3B top middle panel), which was expected. A smaller percentage had better task performance during the more difficult task (29%, Figure 3B top right panel). Some of these participants with improved task performance moved in the expected direction when using the attentional gait index (gray in Figure 3B bottom middle panel), perhaps identifying individuals who improved their performance at a cost of greater PFC recruitment. The increased PFC recruitment could represent a compensatory strategy to cope with the task demand, which corresponds to reduced automaticity.

In summary, as task difficulty increases, more participants showed the expected decrease in automaticity as measured by the attentional gait index and ΔPerformance compared to PFC activation, suggesting that attentional gait index and ΔPerformance are more sensitive to the between task differences within the participants.

### 3.2 Greater attentional gait index is related to worse cognitive function

All independent variables (MMSE score, age, sex, race, and highest level of education) had VIF less than 10 and were all kept in the multiple regression models.

**Figure 4:**
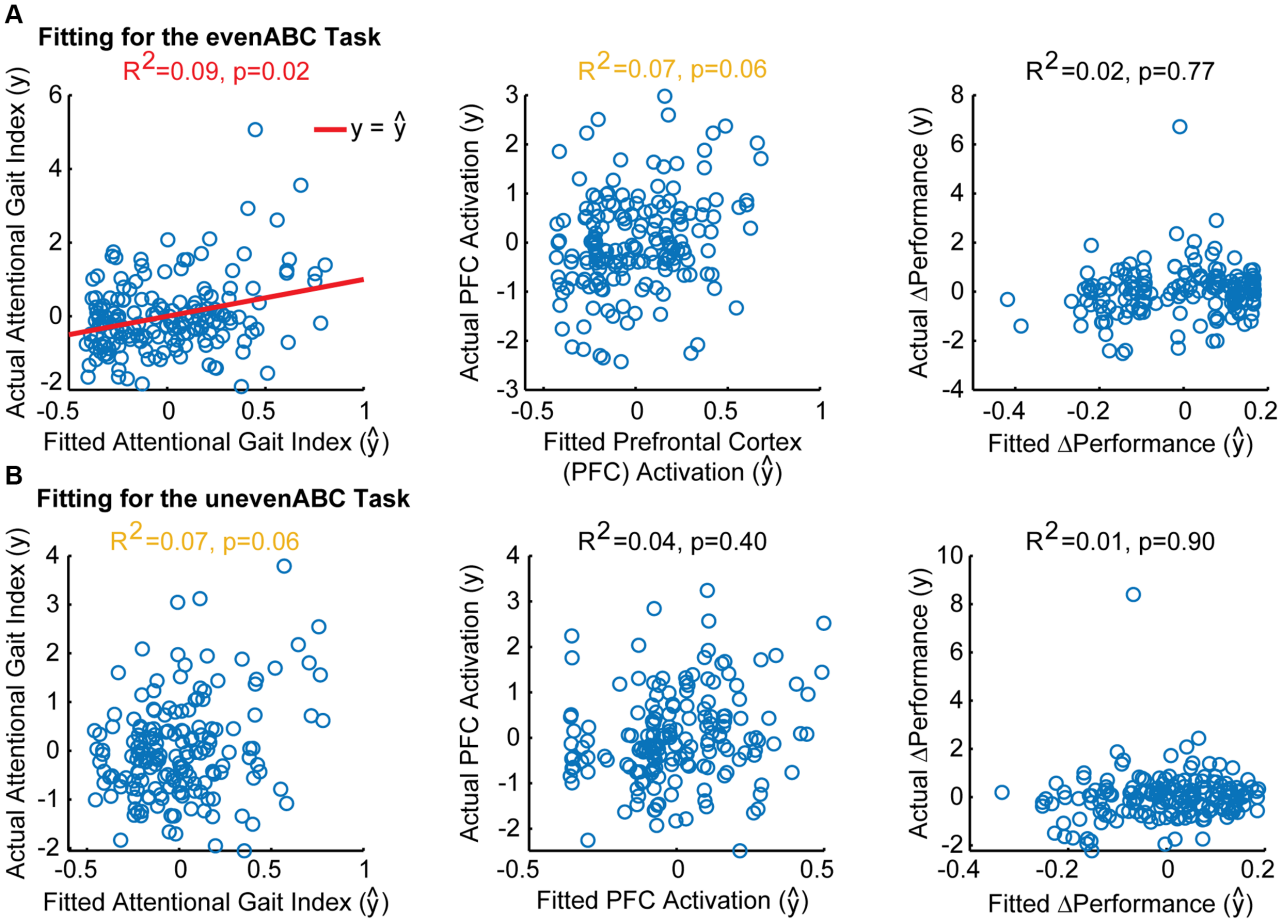
Model fitting between Mini-Mental State Exam (MMSE) scores and attentional gait index, PFC_Activation_, and ΔPerformance after adjusting for age, sex, race, and highest level of education. Actual vs. fitted values for each model in the evenABC task **(A)** and unevenABC task **(B)** are shown. Significant models are shown with a red line for y= ŷ. Trending models are shown in yellow text. A perfect-fitting model would have actual and fitted values following the y= ŷ line. Only the association between MMSE and attentional gait index in the easy task difficulty was significant.

As expected, higher MMSE was associated with lower attentional gait index at both tasks (Table 2; evenABC: *R*^2^ = 0.06, *p* = 0.002; unevenABC: *R*^2^ = 0.05, *p* = 0.003), and this association at the easier task was robust to adjustment by age, sex, race, and highest level of education (Table 3; Figure 4; evenABC: *R*^2^ = 0.09, *p* = 0.02). The association between MMSE and attentional gait index was slightly weaker in the adjusted model at the harder task (Table 3; Figure 4; unevenABC: *R*^2^ = 0.07, *p* = 0.06). Higher MMSE was also associated with lower PFC activation at the easier task (Table 2; evenABC: *R*^2^ = 0.03, *p* = 0.02, β=−0.18±0.08 ), but this relation was weaker (Table 3; evenABC: *R*^2^ = 0.07, *p* = 0.06, β=−0.13±0.08 ) after adjusting for covariates.

**Table 2:** Unadjusted regression models of different gait automaticity measures with Mini-Mental State Exam (MMSE) scores (*n* = 173).

Model	*R* ^2^	Model *p*-value	Standardized β_MMSE (Estimates ± SE)	β_MMSE *p*-value
evenABC Task				
Attentional Gait Index_evenABC_	0.06	0.002	−0.23 ± 0.07	0.002
PFCActivation_evenABC_	0.03	0.02	−0.18 ± 0.08	0.02
ΔPerformance_evenABC_	0.005	0.34	0.07 ± 0.08	0.34
unevenABC Task				
Attentional Gait Index_unevenABC_	0.05	0.003	−0.22 ± 0.07	0.003
PFCActivation_unevenABC_	0.02	0.08	−0.13 ± 0.08	0.08
ΔPerformance_unevenABC_	0.005	0.36	0.07 ± 0.08	0.36

Significant models are highlighted in red and trending (*p* < 0.1) models are shown in yellow.

**Table 3:** Multivariable regression models of different metrics to quantify gait automaticity with Mini-Mental State Exam (MMSE) scores (*n* = 173) adjusted for age, sex, race, and highest level of education.

Model	*R* ^2^	Model *p*-value	Standardized β_MMSE (Estimates ± SE)	β_MMSE *p*-value
evenABC Task				
Attentional Gait Index_evenABC_	0.09	0.02	−0.20 ± 0.08	0.01
PFCActivation_evenABC_	0.07	0.06	−0.13 ± 0.08	0.10
ΔPerformance_evenABC_	0.02	0.77	0.08 ± 0.08	0.30
unevenABC Task				
Attentional Gait Index_unevenABC_	0.07	0.06	−0.19 ± 0.08	0.02
PFCActivation_unevenABC_	0.04	0.40	−0.10 ± 0.08	0.21
ΔPerformance_unevenABC_	0.01	0.90	0.07 ± 0.08	0.41

Significant models are highlighted in red, and trending (*p* < 0.1) models are highlighted in yellow.

The association between MMSE and attentional gait index had the largest variance accounted for and largest standardized β magnitude across both tasks in the unadjusted (Table 2; even ABC: β=−0.23±0.07,p=0.002 ; unevenABC: β=−0.22±0.07,p=0.003 ) and adjusted (Table 3; even ABC: β=−0.20±0.08,p=0.01 ; unevenABC: β=−0.19±0.08,p=0.02 ) models.

The result remains robust after removing the two participants who did not follow instructions to recite every other letter of the alphabet. The models between MMSE and attentional gait index still had the largest variance accounted for and largest standardized β magnitude across both tasks (Task evenABC: Attentional Gait Index *R*^2^ = 0.10, β=−0.21 , PFC_Activation_: *R*^2^ = 0.07, β=−0.14 , ΔPerformance: *R*^2^ = 0.03, β = 0.13. Task unevenABC: Attentional Gait Index R^2^ = 0.08, β=−0.20, PFC_Activation_: R^2^ = 0.04, β=−0.10, ΔPerformance: *R*^2^ = 0.05, β = 0.13).

In sum, the MMSE score was more strongly correlated with the attentional gait index than with PFC activation or performance. The association between MMSE scores and attentional gait index had the largest standardized β magnitude and variance explained across both DT difficulties. The result is robust to adjustments for covariates and not sensitive to the removal of participants who did not follow instructions.

## 4 Discussion

We combined PFC activation and DT performance measures to create a monotonic index to quantify gait automaticity. We showed that the attentional gait index better captured the task difficulty-related change in automaticity and was more strongly related to general cognitive function than either PFC activation or DT performance alone. In summary, the proposed index was effective at differentiating (1) between tasks and (2) between participants.

### 4.1 Task performance contributes valuable information to the attentional gait index when comparing task difficulties

When comparing task difficulties, we expected the harder task to show worse automaticity; specifically, the more challenging task will require increased attentional demand from the PFC and result in decreased performance due to greater interference between the motor and cognitive tasks. This expected change would be reflected as an increase in the attentional gait index, an increase in PFC activation, and a decrease in performance. We observed that 73% of participants increased attentional gait index and 71% decreased performance as expected, but only 57% of participants increased PFC activation from evenABC to unevenABC. The result confirms that the unevenABC task was more challenging to the participants, given the majority of the participants decreased performance. However, this increased challenge was not always reflected in PFC activation alone.

Multiple reasons could explain why participants did not increase PFC activation with increasing task demands: inefficient recruitment of necessary neural resources, the task exceeding capacity (Reuter-Lorenz and Cappell, 2008), or disengagement from the task such that the participant did not even try to cope with the difficulty. In addition to individual differences, the inconsistency in the PFC activity could also be attributed to measurement noise from fNIRS, such as physiological changes and skin properties (Vitorio et al., 2017; Menant et al., 2020). This observation emphasizes the heterogeneity of PFC response despite consistent behavioral performance and the importance of incorporating behavioral performance when interpreting PFC activity.

Our finding that DT performance contains crucial information when assessing automaticity aligns with previous studies showing that DT performance is related to older adults’ mobility (Montero-Odasso et al., 2012; Verghese et al., 2012; Rosso et al., 2019a) and cognitive abilities (Holtzer et al., 2006; Rosso et al., 2019b; Brauner et al., 2021). Nevertheless, performance alone does not quantify automaticity since automaticity is defined by proficient performance alongside minimal neural inputs from the attentional and executive control center (Clark, 2015). In other words, the engagement of attentional resources during a typically automatic task such as walking is a signature of reduced automaticity (Van Swearingen and Studenski, 2014; Clark, 2015). Moreover, most theoretical models emphasize the importance of examining the activation patterns of PFC to understand cognitive aging (Reuter-Lorenz and Cappell, 2008; Park and Reuter-Lorenz, 2009; Festini et al., 2018). Considering fNIRS is a relatively new technology with fast-evolving instrument design and analysis techniques (Perrey, 2014), future studies should keep considering performance and PFC activity together using the attentional gait index to improve quantification of gait automaticity.

### 4.2 Poorer attentional gait index was related to lower general cognitive function

When comparing across participants, we showed that lower cognitive function, measured by MMSE score, is related to lower attentional gait index in both DT difficulties. The results suggest that the attentional gait index was more sensitive to individual characteristics that could impact automaticity than PFC activation or performance, which are existing metrics to quantify automaticity.

However, we noticed that the variance explained by the models (*R*^2^) is relatively low. The fit remains roughly the same with or without adjusting for age, sex, race, and highest level of education, suggesting that most of the variance in the attentional gait index and PFC activity was not explained by the demographics and cognitive abilities. The low *R*^2^ is not unexpected as gait automaticity depends on the subcortical circuits, which were not measured with fNIRS during walking (Wu and Hallett, 2005; Rosano et al., 2007; Clark, 2015). Thus, complementary imaging data about the integrity of the subcortical circuits, including volume or circuitry connectivity, might account for the unexplained variance in the model.

In our study, MMSE was not related to ΔPerformance. In comparison, a prior study (Brauner et al., 2021) has found that the MMSE score is associated with ΔPerformance. Of note, ΔPerformance is a component in calculating the attentional gait index, but we were not able to observe a direct association between MMSE score and ΔPerformance in our study. Several factors could contribute to this difference, including (1) a different dual-task paradigm was used and (2) the population recruited might be different. Specifically, the prior study had an older cohort than we did ( ≥ 85 years old vs. mean 73.9 ± 5.6 in our sample).

### 4.3 Attentional gait index provides a unified measure of gait automaticity that combines behavioral performance and PFC activation

Performance in DT walking is a commonly used metric to assess gait automaticity, but performance alone does not reflect the cortical input required. For instance, the same performance can be achieved with little effort and low attentional control, i.e., automaticity, or with significant effort from the attentional resource center. Neurophysiological measures, such as PFC activation, provide more insights into the cortical inputs supporting the observed performance. However, prior research on PFC signals during walking has shown inconsistent results (Yeung and Chan, 2021). Both increased (Holtzer et al., 2011; Mirelman et al., 2017) and decreased (Beurskens et al., 2014) PFC activity during DT walking have been reported. These findings are hard to reconcile without considering task performance.

The attentional gait index provides a solution by establishing a unified axis that combines performance and PFC activation. The increased PFC activity observed by Holtzer et al. (2011) and Mirelman et al. (2017) could indicate either increased reliance on the attentional center to compensate for the loss of automaticity, resulting in maintained task performance or dedifferentiation in the neural signals, leading to poor task performance. In the attentional gait index, the increased PFC activity would be scaled down for maintained performance to reflect successful compensation and scaled up for poor task performance to reflect unsuccessful compensation or dedifferentiation. The decreased PFC activity reported by Beurskens et al. (2014) could represent either inefficiency of resource utilization, leading to poor task performance, or maintained automaticity, resulting in good performance. These differences would be reflected in the attentional gait index, where the same PFC activity would be scaled up for the reduced performance scenario to reflect the inability to recruit necessary resources.

## 5 Limitations

One main limitation is that the attentional gait index can only be computed for study designs with at least two distinct task difficulties because parameter optimization α requires at least two difficulties. Additionally, even though the proposed method to compute the attentional gait index can be applied to any dual-task paradigm with at least two levels of task difficulties, the datasets used to test the index here all performed the same dual-task walking paradigm (i.e., walking and reciting alternating letters of the alphabet with or without an uneven surface) with no task prioritization. It has been shown that dual-task modality and prioritization can impact performance and assessment results differently (Verghese et al., 2007; Yogev-Seligmann et al., 2010; Beurskens et al., 2014; Laguë-Beauvais et al., 2015; Tsang et al., 2022). Therefore, it is important to verify that the index design is robust to varying experimental protocols, including different motor and cognitive tasks, prioritization instructions, and performance metrics, in future studies. Finally, the participants recruited are relatively healthy in their cognitive abilities (MMSE mean ± SD: 28.42 ± 1.83, min = 21), which may not reflect the full population of community-dwelling older adults (Jacqmin-Gadda et al., 1997). Future studies can assess if the findings are generalizable to populations with a wider range of cognitive abilities. Future studies should also investigate if the index is related to clinically relevant measures such as balance confidence or fall risks.

## 6 Conclusion

Gait automaticity is crucial for safe community mobility, and automaticity is an important rehabilitation target to restore walking function and independence. However, a standard and robust assessment for gait automaticity is lacking. We addressed the need to better quantify gait automaticity using a novel approach to combine both DT performance and PFC activation into an attentional gait index. We demonstrated the efficacy of the index by achieving two objectives, specifically, better-differentiating automaticity (1) between tasks within participants and (2) between participants based on overall cognitive function. The index revealed a decrease in automaticity as task difficulty increased, which was not evidenced by PFC activation. Furthermore, the index captured the cognitive ability-related differences in automaticity better than PFC activation or DT performance alone. In summary, the attentional gait index provided a standard metric to characterize gait automaticity in a dual-task walking paradigm. The standardized metric will allow better quantification of the effectiveness of interventions aimed at improving automaticity and facilitate future studies to investigate the connections between automaticity and other participant-specific characteristics.

## Data availability statement

The data analyzed in this study is subject to the following licenses/restrictions: The raw data supporting the conclusions of the article will be made available by the authors upon request. Requests to access these datasets should be directed to SL, shl187@pitt.edu; AR, ALR143@pitt.edu; GT-O, gelsyto@pitt.edu.

## Ethics statement

The studies involving humans were approved by the Institutional Review Board at the University of Pittsburgh. The studies were conducted in accordance with the local legislation and institutional requirements. The participants provided their written informed consent to participate in this study.

## Author contributions

SL: Conceptualization, Data curation, Formal analysis, Methodology, Software, Visualization, Writing – original draft, Writing – review & editing. AR: Conceptualization, Funding acquisition, Methodology, Project administration, Resources, Supervision, Writing – review & editing. EB: Conceptualization, Data curation, Methodology, Validation, Writing – review & editing. AW: Conceptualization, Supervision, Writing – review & editing. CR: Writing – review & editing, Funding acquisition, Project administration, Resources. GT-O: Conceptualization, Funding acquisition, Methodology, Project administration, Resources, Supervision, Writing – review & editing.

## References

[ref1] Aizenstein H. J. Nebes R. D. Saxton J. A. Price J. C. Mathis C. A. Tsopelas N. D.. (2008). Frequent amyloid deposition without significant cognitive impairment among the elderly. Arch. Neurol. 65, 1509–1517. doi: 10.1001/archneur.65.11.1509, PMID: 19001171 PMC2636844

[ref2] Barker J. W. Aarabi A. Huppert T. J. (2013). Autoregressive model based algorithm for correcting motion and serially correlated errors in fNIRS. Biomed. Opt. Express 4, 1366–1379. doi: 10.1364/boe.4.001366, PMID: 24009999 PMC3756568

[ref3] Beurskens R. Helmich I. Rein R. Bock O. (2014). Age-related changes in prefrontal activity during walking in dual-task situations: a fNIRS study. Int. J. Psychophysiol. 92, 122–128. doi: 10.1016/j.ijpsycho.2014.03.005, PMID: 24681355

[ref4] Bohlke K. Perera S. Baillargeon E. M. Redfern M. S. Sparto P. J. Sejdic E.. (2023). Exercise interventions, postural control, and prefrontal cortex activation in older adults. Brain Cogn. 171:106063. doi: 10.1016/j.bandc.2023.106063, PMID: 37523831 PMC10529535

[ref5] Brach J. S. Van Swearingen J. M. Gil A. Nadkarni N. K. Kriska A. Cham R.. (2020). Program to improve mobility in aging (PRIMA) study: methods and rationale of a task-oriented motor learning exercise program. Contemp. Clin. Trials 89:105912. doi: 10.1016/j.cct.2019.105912, PMID: 31838258 PMC6945812

[ref6] Brandler T. C. Oh-Park M. Wang C. Holtzer R. Verghese J. (2012). Walking while talking: investigation of alternate forms. Gait Posture 35, 164–166. doi: 10.1016/j.gaitpost.2011.08.003, PMID: 21944476 PMC3296479

[ref7] Brauner F. O. Balbinot G. Figueiredo A. I. Hausen D. O. Schiavo A. Mestriner R. G. (2021). The performance index identifies changes across the dual task timed up and go test phases and impacts task-cost estimation in the oldest-old. Front. Hum. Neurosci. 15, 1–12. doi: 10.3389/fnhum.2021.720719, PMID: 34658817 PMC8514992

[ref8] Brustio P. R. Rabaglietti E. Formica S. Liubicich M. E. (2018). Dual-task training in older adults: the effect of additional motor tasks on mobility performance. Arch. Gerontol. Geriatr. 75, 119–124. doi: 10.1016/j.archger.2017.12.003 29245071

[ref9] Cabeza R. (2002). Hemispheric asymmetry reduction in older adults: the HAROLD model. Psychol. Aging 17, 85–100. doi: 10.1037/0882-7974.17.1.85 11931290

[ref10] Cabeza R. Dennis N. A. (2012). “Frontal lobes and aging” in Principles of frontal lobe function. eds. Stuss D. T. Knight R. T. (Oxford: Oxford University Press), 628–652.

[ref11] Clark D. J. (2015). Automaticity of walking: functional significance, mechanisms, measurement and rehabilitation strategies. Front. Hum. Neurosci. 9, 1–13. doi: 10.3389/fnhum.2015.00246, PMID: 25999838 PMC4419715

[ref12] Clark D. J. Christou E. A. Ring S. A. Williamson J. B. Doty L. (2014a). Enhanced somatosensory feedback reduces prefrontal cortical activity during walking in older adults. J. Gerontol. A Biol. Sci. Med. Sci. 69, 1422–1428. doi: 10.1093/gerona/glu125, PMID: 25112494 PMC4229993

[ref13] Clark D. J. Rose D. K. Ring S. A. Porges E. C. (2014b). Utilization of central nervous system resources for preparation and performance of complex walking tasks in older adults. Front. Aging Neurosci. 6, 1–9. doi: 10.3389/fnagi.2014.00217, PMID: 25202270 PMC4142860

[ref14] Festini S. B. Zahodne L. Reuter-Lorenz P. A. (2018). Theoretical perspectives on age differences in brain activation: harold, pasa, crunch—how do they STAC up? Oxford University Press: Oxford

[ref15] Fettrow T. Hupfeld K. Tays G. Clark D. J. Reuter-Lorenz P. A. Seidler R. D. (2021). Brain activity during walking in older adults: implications for compensatory versus dysfunctional accounts. Neurobiol. Aging 105, 349–364. doi: 10.1016/j.neurobiolaging.2021.05.015, PMID: 34182403 PMC8338893

[ref16] Folstein M. F. Folstein S. E. McHugh P. R. (1975). “Mini-mental state”. A practical method for grading the cognitive state of patients for the clinician. J. Psychiatr. Res. 12, 189–198. doi: 10.1016/0022-3956(75)90026-6 1202204

[ref17] Fraser S. A. Dupuy O. Pouliot P. Lesage F. Bherer L. (2016). Comparable cerebral oxygenation patterns in younger and older adults during dual-task walking with increasing load. Front. Aging Neurosci. 8, 1–9. doi: 10.3389/fnagi.2016.00240, PMID: 27812334 PMC5071361

[ref18] Ganguli M. Chang C. C. H. Snitz B. E. Saxton J. A. Vanderbilt J. Lee C. W. (2010). Prevalence of mild cognitive impairment by multiple classifications: the Monongahela-youghiogheny healthy aging team (MYHAt) project. Am. J. Geriatr. Psychiatr. 18, 674–683. doi: 10.1097/JGP.0b013e3181cdee4f, PMID: 20220597 PMC2906673

[ref19] Gschwind Y. J. Bridenbaugh S. A. Kressig R. W. (2010). Gait disorders and falls. GeroPsych 23, 21–32. doi: 10.1024/1662-9647/a000004

[ref20] Herold F. Wiegel P. Scholkmann F. Thiers A. Hamacher D. Schega L. (2017). Functional near-infrared spectroscopy in movement science: a systematic review on cortical activity in postural and walking tasks. Neurophotonics 4:041403. doi: 10.1117/1.nph.4.4.041403, PMID: 28924563 PMC5538329

[ref21] Holtzer R. Mahoney J. R. Izzetoglu M. Izzetoglu K. Onaral B. Verghese J. (2011). fNIRS study of walking and walking while talking in young and old individuals. J. Gerontol. A Biol. Sci. Med. Sci. 66, 879–887. doi: 10.1093/gerona/glr068, PMID: 21593013 PMC3148759

[ref22] Holtzer R. Mahoney J. R. Izzetoglu M. Wang C. England S. Verghese J. (2015). Online fronto-cortical control of simple and attention-demanding locomotion in humans. Neuroimage 112, 152–159. doi: 10.1016/j.neuroimage.2015.03.002, PMID: 25765257 PMC4408246

[ref23] Holtzer R. Verghese J. Xue X. Lipton R. B. (2006). Cognitive processes related to gait velocity: results from the Einstein aging study. Neuropsychology 20, 215–223. doi: 10.1037/0894-4105.20.2.215, PMID: 16594782

[ref24] Hoppes C. W. Huppert T. J. Whitney S. L. Dunlap P. M. Disalvio N. L. Alshebber K. M.. (2020). Changes in cortical activation during dual-task walking in individuals with and without visual Vertigo. J. Neurol. Phys. Ther. 44, 156–163. doi: 10.1097/NPT.0000000000000310, PMID: 32168158 PMC7112165

[ref25] Jacqmin-Gadda H. Fabrigoule C. Commenges D. Dartigues J. F. (1997). A 5-year longitudinal study of the mini-mental state examination in normal aging. Am. J. Epidemiol. 145, 498–506. doi: 10.1093/oxfordjournals.aje.a009137 9063339

[ref26] Laguë-Beauvais M. Fraser S. A. Desjardins-Crépeau L. Castonguay N. Desjardins M. Lesage F.. (2015). Shedding light on the effect of priority instructions during dual-task performance in younger and older adults: a fNIRS study. Brain Cogn. 98, 1–14. doi: 10.1016/j.bandc.2015.05.001 26046834

[ref27] Leys C. Ley C. Klein O. Bernard P. Licata L. (2013). Detecting outliers: do not use standard deviation around the mean, use absolute deviation around the median. J. Exp. Soc. Psychol. 49, 764–766. doi: 10.1016/j.jesp.2013.03.013

[ref28] Longhurst J. K. Rider J. V. Cummings J. L. John S. E. Poston B. Held Bradford E. C.. (2022). A novel way of measuring dual-task interference: the reliability and construct validity of the dual-task effect battery in neurodegenerative disease. Neurorehabil. Neural Repair 36, 346–359. doi: 10.1177/15459683221088864, PMID: 35387509 PMC9133058

[ref29] Maillet D. Rajah M. N. (2013). Association between prefrontal activity and volume change in prefrontal and medial temporal lobes in aging and dementia: a review. Ageing Res. Rev. 12, 479–489. doi: 10.1016/j.arr.2012.11.001, PMID: 23183352

[ref30] Menant J. C. Maidan I. Alcock L. Al-Yahya E. Cerasa A. Clark D. J.. (2020). A consensus guide to using functional near-infrared spectroscopy in posture and gait research. Gait Posture 82, 254–265. doi: 10.1016/j.gaitpost.2020.09.012, PMID: 32987345

[ref31] Mirelman A. Maidan I. Bernad-Elazari H. Shustack S. Giladi N. Hausdorff J. M. (2017). Effects of aging on prefrontal brain activation during challenging walking conditions. Brain Cogn. 115, 41–46. doi: 10.1016/j.bandc.2017.04.002 28433922

[ref32] Miyai I. Tanabe H. C. Sase I. Eda H. Oda I. Konishi I.. (2001). Cortical mapping of gait in humans: a near-infrared spectroscopic topography study. Neuroimage 14, 1186–1192. doi: 10.1006/nimg.2001.0905, PMID: 11697950

[ref33] Montero-Odasso M. Muir S. W. Hall M. Doherty T. J. Kloseck M. Beauchet O.. (2011). Gait variability is associated with frailty in community-dwelling older adults. J. Gerontol. A Biol. Sci. Med. Sci. 66, 568–576. doi: 10.1093/gerona/glr007 21357190

[ref34] Montero-Odasso M. Verghese J. Beauchet O. Hausdorff J. M. (2012). Gait and cognition: a complementary approach to understanding brain function and the risk of falling. J. Am. Geriatr. Soc. 60, 2127–2136. doi: 10.1111/j.1532-5415.2012.04209.x, PMID: 23110433 PMC3498517

[ref35] Osofundiya O. Benden M. E. Dowdy D. Mehta R. K. (2016). Obesity-specific neural cost of maintaining gait performance under complex conditions in community-dwelling older adults. Clin. Biomech. 35, 42–48. doi: 10.1016/j.clinbiomech.2016.03.011, PMID: 27124085

[ref36] Park D. C. Reuter-Lorenz P. (2009). The adaptive brain: aging and neurocognitive scaffolding. Annu. Rev. Psychol. 60, 173–196. doi: 10.1146/annurev.psych.59.103006.093656, PMID: 19035823 PMC3359129

[ref37] Patla A. E. Shumway-Cook A. (1999). Dimensions of mobility: defining the complexity and difficulty associated with community mobility. J. Aging Phys. Act. 7, 7–19. doi: 10.1123/japa.7.1.7

[ref38] Paul S. S. Ada L. Canning C. G. (2005). Automaticity of walking–implications for physiotherapy practice. Phys. Ther. Rev. 10, 15–23. doi: 10.1179/108331905X43463

[ref39] Perrey S. (2014). Possibilities for examining the neural control of gait in humans with fNIRS. Front. Physiol. 5, 10–13. doi: 10.3389/fphys.2014.00204, PMID: 24904433 PMC4035560

[ref40] Rantakokko M. Portegijs E. Viljanen A. Iwarsson S. Kauppinen M. Rantanen T. (2016). Changes in life-space mobility and quality of life among community-dwelling older people: a 2-year follow-up study. Qual. Life Res. 25, 1189–1197. doi: 10.1007/s11136-015-1137-x, PMID: 26407605

[ref41] Reuter-Lorenz P. A. Cappell K. A. (2008). Neurocognitive aging and the compensation hypothesis. Curr. Dir. Psychol. Sci. 17, 177–182. doi: 10.1111/j.1467-8721.2008.00570.x

[ref42] Rosano C. Aizenstein H. J. Studenski S. Newman A. B. (2007). A regions-of-interest volumetric analysis of mobility limitations in community-dwelling older adults. J. Gerontol. A Biol. Sci. Med. Sci. 62, 1048–1055. doi: 10.1093/gerona/62.9.1048, PMID: 17895446

[ref43] Rosso A. L. Metti A. L. Faulkner K. Brach J. S. Studenski S. A. Redfern M.. (2019a). Associations of usual pace and complex task gait speeds with incident mobility disability. J. Am. Geriatr. Soc. 67, 2072–2076. doi: 10.1111/jgs.16049, PMID: 31318048 PMC6800783

[ref44] Rosso A. L. Metti A. L. Faulkner K. Redfern M. Yaffe K. Launer L.. (2019b). Complex walking tasks and risk for cognitive decline in high functioning older adults. J. Alzheimers Dis. 71, S65–S73. doi: 10.3233/JAD-181140, PMID: 30814353 PMC6703970

[ref45] Rosso A. L. Taylor J. A. Tabb L. P. Michael Y. L. (2013). Mobility, disability, and social engagement in older adults. J. Aging Health 25, 617–637. doi: 10.1177/0898264313482489, PMID: 23548944 PMC3683993

[ref46] Santosa H. Zhai X. Fishburn F. Huppert T. (2018). The NIRS brain Analyz IR toolbox. Algorithms 11:73. doi: 10.3390/A11050073 PMC11218834 38957522

[ref47] Sheppard K. D. Sawyer P. Ritchie C. S. Allman R. M. Brown C. J. (2013). Life-space mobility predicts nursing home admission over 6 years. J. Aging Health 25, 907–920. doi: 10.1177/0898264313497507, PMID: 23965310 PMC4071297

[ref48] Teng E. L. Chui H. C. (1987). The modified Mini-mental state (3MS) examination. J. Clin. Psychiatry 48, 314–318. 3611032

[ref49] Thies S. B. Richardson J. K. DeMott T. Ashton-Miller J. A. (2005). Influence of an irregular surface and low light on the step variability of patients with peripheral neuropathy during level gait. Gait Posture 22, 40–45. doi: 10.1016/j.gaitpost.2004.06.006, PMID: 15996590

[ref50] Tsang C. S. L. Wang S. Miller T. Pang M. Y. C. (2022). Degree and pattern of dual-task interference during walking vary with component tasks in people after stroke: a systematic review. J. Physiother. 68, 26–36. doi: 10.1016/j.jphys.2021.12.009, PMID: 34953757

[ref51] Van Patten R. Britton K. Tremont G. (2019). Comparing the Mini-mental state examination and the modified Mini-mental state examination in the detection of mild cognitive impairment in older adults. Int. Psychogeriatr. 31, 693–701. doi: 10.1017/S1041610218001023, PMID: 30021667

[ref52] Van Swearingen J. M. Studenski S. A. (2014). Aging, motor skill, and the energy cost of walking: implications for the prevention and treatment of mobility decline in older persons. J. Gerontol. A Biol. Sci. Med. Sci. 69, 1429–1436. doi: 10.1093/gerona/glu153, PMID: 25182600 PMC4271095

[ref53] Verghese J. Holtzer R. Lipton R. B. Wang C. (2012). Mobility stress test approach to predicting frailty, disability, and mortality in high-functioning older adults. J. Am. Geriatr. Soc. 60, 1901–1905. doi: 10.1111/j.1532-5415.2012.04145.x, PMID: 23002714 PMC3470773

[ref54] Verghese J. Kuslansky G. Holtzer R. Katz M. Xue X. Buschke H.. (2007). Walking while talking: effect of task prioritization in the elderly. Arch. Phys. Med. Rehabil. 88, 50–53. doi: 10.1016/j.apmr.2006.10.007, PMID: 17207675 PMC1894901

[ref55] Vitorio R. Stuart S. Rochester L. Alcock L. Pantall A. (2017). fNIRS response during walking — artefact or cortical activity? A systematic review. Neurosci. Biobehav. Rev. 83, 160–172. doi: 10.1016/j.neubiorev.2017.10.002 29017917

[ref56] Wu T. Hallett M. (2005). The influence of normal human ageing on automatic movements. J. Physiol. 562, 605–615. doi: 10.1113/jphysiol.2004.076042, PMID: 15513939 PMC1665504

[ref57] Yeung M. K. Chan A. S. (2021). A systematic review of the application of functional near-infrared spectroscopy to the study of cerebral hemodynamics in healthy aging. Neuropsychol. Rev. 31, 139–166. doi: 10.1007/s11065-020-09455-3 32959167

[ref58] Yogev-Seligmann G. Rotem-Galili Y. Mirelman A. Dickstein R. Giladi N. Hausdorff J. M. (2010). How does explicit prioritization alter walking during dual-task performance? Effects of age and sex on gait speed and variability. Phys. Ther. 90, 177–186. doi: 10.2522/ptj.20090043, PMID: 20023000 PMC2816029

