# Supplementary material for: Novel attentional gait index reveals a cognitive ability-related decline in gait automaticity during dual-task walking
Source: Front Aging Neurosci. 2024 Jan 11;15:1283376. doi: 10.3389/fnagi.2023.1283376 (PMC10808635; doi:10.3389/fnagi.2023.1283376)
Supplement: Supplementary file 1 [file Data_Sheet_1.docx]

Supplementary Material

# Calculating the attentional gait index with ΔHbr

Typically, when neural activity increases, Hbo increases and Hbr decreases locally, resulting in a positive $\Delta H$bo and negative $\Delta H$br during the task compared to rest. Given this, if we were to use Hbr to represent the PFC activations, Hbr is first sign flipped to maintain the convention where higher $PFC_{activation}$ always represents more PFC neural activity, specifically:

$$If using Hbr, PFC_{activation}=f\left( -\Delta Hbr \right)$$

$$=-\Delta Hbr+\min\left( -\Delta Hbr \right)+\varepsilon$$

$$\varepsilon>0$$

All other steps to calculate the attentional gait index remain the same.

# Supplementary Figures and Tables

## Supplementary Figures


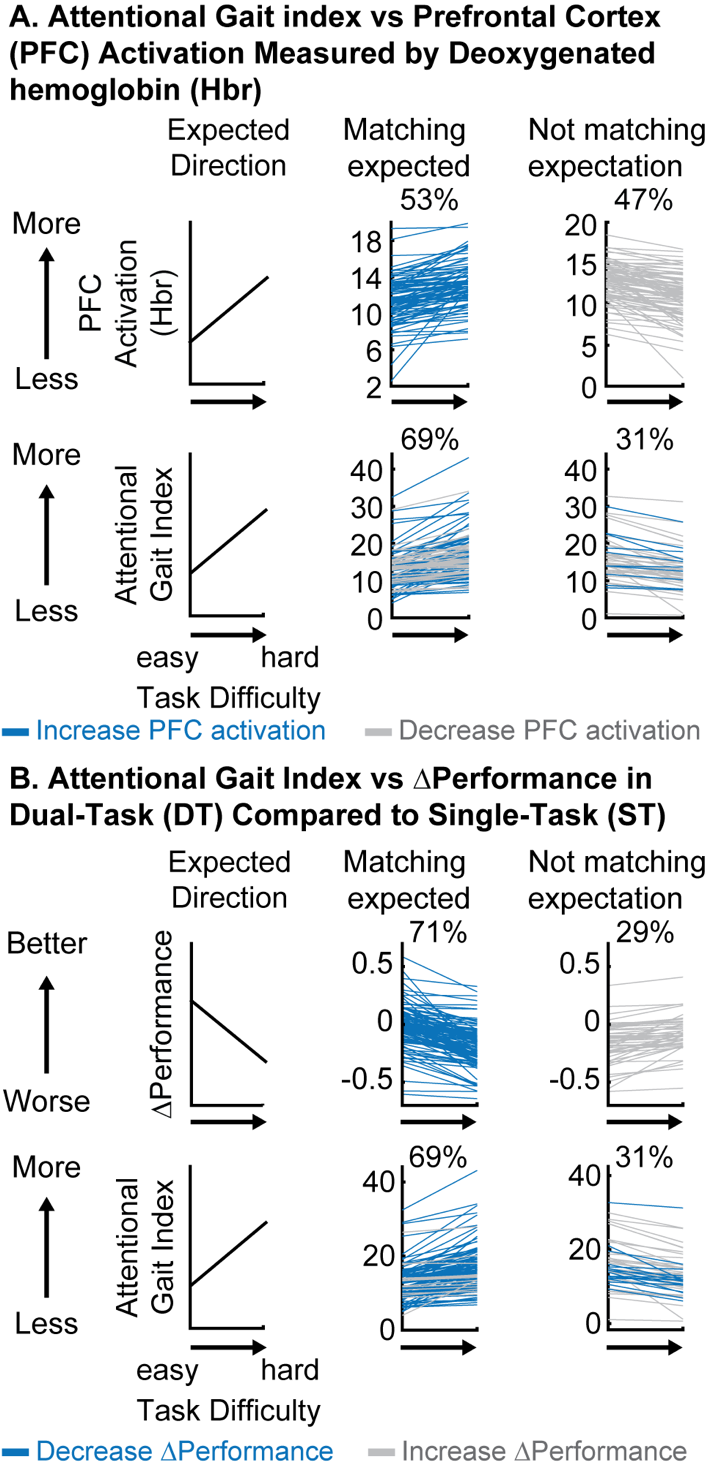
**Supplementary Figure 1. Descriptive comparison between attentional gait index (computed with Hbr), PFC activation (measured by Hbr), and** $\boldsymbol{\Delta}$**Performance as task difficulty increases.** Overall, the result remains consistent with the ones from Hbo. Both the index and $\Delta$Performance are sensitive to decrease in automaticity as task difficulty increases, whereas the PFC activation shows no clear differentiation between tasks. **(A)** Comparison between attentional gait index and PFC activation at each dual-task (DT) relative to rest. Blue always represents participants who showed an expected increase in PFC activation (top middle panel, notice that the Hbr has been sign flipped and shifted to above 0 such that higher value represents more PFC activity) and grey always represents participants who decreased PFC activation (top right) as the task became more difficult. Colors in the bottom panel represent how participants from different groups at the top panel moved into different categories (increase or decrease) in attentional gait index. Notice that some participants with an unexpected decrease in PFC activation will now have an expected increase in attentional gait index after considering performance (grey in the top right moved to the bottom middle panel). **(B)** Comparison between attentional gait index and $\Delta$Performance. Blue always represents participants who showed an expected decrease in $\Delta$Performance as the task becomes harder (top middle panel) and grey always represents the ones who increased $\Delta$Performance (top right). Colors in the bottom panel represent how participants from different $\Delta$Performance at the top panel moved into different categories (increase or decrease) in attentional gait index (bottom panel).

## Supplementary Tables

## Supplementary Table 1. Prefrontal cortical (PFC) activation as measured by deoxygenated hemoglobin (Hbr) of the study participants. Data are shown as mean$\boldsymbol{\pm}$SD.

| **Variables** | **Mean (SD)** |
| --- | --- |
| Change in deoxygenated hemoglobin ($\Delta$Hbr) from rest to evenABC (t-stats) | -0.55 ± 2.53 |
| $\Delta H$br from rest to unevenABC (t-stats) | -0.54 ± 2.81 |

**Supplementary Table 2. Unadjusted regression models of different gait automaticity measures (attentional gait index computed from Hbr, PFCActivation measured by Hbr, and** $\boldsymbol{\Delta}$**Performance) with Mini-Mental Sate Exam (MMSE) score (n=173).** Significant models are highlighted in red. Notice that the models with $\Delta$Performance are the same as the ones in Table 2 of the main text. Among the two significant models, the model between MMSE and attentional gait index at evenABC had non normal residuals, violating the assumptions of linear regression. The only valid and significant model is between MMSE and the index at unevenABC. This model also had the largest variance accounted for and largest standardized $\beta$ magnitude.

| **Model** | **R^2^** | **Model**  **p-value** | **Standardized** $\boldsymbol{\beta}_{\mathbf{MMSE}}$  **(Estimates** $\boldsymbol{\pm}$ **SE)** | $\boldsymbol{\beta}_{\mathbf{MMSE}}$ **p-value** |
| --- | --- | --- | --- | --- |
| **evenABC Task** |  |  |  |  |
| Attentional Gait Index_evenABC_ | 0.03 | 0.02 | -0.18 $\pm$ 0.08 | 0.02 |
| PFCActivation_evenABC_ | 0.01 | 0.24 | -0.09 $\pm$0.08 | 0.24 |
| $\Delta$Performance_evenABC_ | 0.005 | 0.34 | 0.07 $\pm$ 0.08 | 0.34 |
| **unevenABC Task** |  |  |  |  |
| Attentional Gait Index_unevenABC_ | 0.04 | 0.01 | -0.21 $\pm$0.07 | 0.01 |
| PFCActivation_unevenABC_ | 0.01 | 0.20 | -0.10 $\pm$ 0.08 | 0.20 |
| $\Delta$Performance_unevenABC_ | 0.005 | 0.36 | 0.07 $\pm$ 0.08 | 0.36 |

**Supplementary Table 3. Multivariable regression models of different metrics to quantify gait automaticity (attentional gait index computed from Hbr, PFCActivation measured by Hbr, and** $\boldsymbol{\Delta}$**Performance) with Mini-Mental State Exam (MMSE) scores (n=173) adjusted for age, sex, race, and highest level of education.** Significant models are highlighted in red. Notice that the models with $\Delta$Performance are the same as the ones in Table 3 of the main text. Similar to the result using Hbo in the main text, the models between MMSE and the index remain significant after adjusting for demographic variables and had largest variance accounted for as well as largest standardized $\beta$ magnitude. Residuals were also normal for both models between MMSE and attentional gait index.

| **Model** | **R^2^** | **Model**  **p-value** | **Standardized**$\boldsymbol{\beta}_{\mathbf{MMSE}}$  **(Estimates** $\boldsymbol{\pm}$ **SE)** | $\boldsymbol{\beta}_{\mathbf{MMSE}}$ **p-value** |
| --- | --- | --- | --- | --- |
| **evenABC Task** |  |  |  |  |
| Attentional Gait Index_evenABC_ | 0.07 | 0.04 | -0.16 $\pm$0.08 | 0.04 |
| PFCActivation_evenABC_ | 0.06 | 0.15 | -0.06$\pm$ 0.08 | 0.48 |
| $\Delta$Performance_evenABC_ | 0.02 | 0.77 | 0.08 $\pm$ 0.08 | 0.30 |
| **unevenABC Task** |  |  |  |  |
| Attentional Gait Index_unevenABC_ | 0.08 | 0.03 | -0.19 $\pm$ 0.08 | 0.02 |
| PFCActivation_unevenABC_ | 0.06 | 0.11 | -0.07 $\pm$0.08 | 0.37 |
| $\Delta$Performance_unevenABC_ | 0.01 | 0.90 | 0.07 $\pm$ 0.08 | 0.41 |
